# Effects of Limited and Extended Pavlovian Training on Devaluation Sensitivity of Sign- and Goal-Tracking Rats

**DOI:** 10.3389/fnbeh.2020.00003

**Published:** 2020-02-04

**Authors:** Sara E. Keefer, Sam Z. Bacharach, Daniel E. Kochli, Jules M. Chabot, Donna J. Calu

**Affiliations:** ^1^Department of Anatomy and Neurobiology, University of Maryland School of Medicine, Baltimore, MD, United States; ^2^Program in Neuroscience, University of Maryland School of Medicine, Baltimore, MD, United States; ^3^Neuroscience and Behavior Program, Wesleyan University, Middletown, CT, United States

**Keywords:** behavioral flexibility, goal-tracking, sign-tracking, outcome devaluation, pavlovian incentive learning, reward

## Abstract

Individual differences in Pavlovian approach predict differences in devaluation sensitivity. Recent studies indicate goal-tracking (GT) rats are sensitive to outcome devaluation while sign-tracking (ST) rats are not. With extended training in Pavlovian lever autoshaping (PLA), GT rats display more lever-directed behavior, typical of ST rats, suggesting they may become insensitive to devaluation with more Pavlovian training experience. Here, we use a within-subject satiety-induced outcome devaluation procedure to test devaluation sensitivity after limited and extended PLA training in GT and ST rats. We trained rats in PLA to determine GT and ST groups. Then, we sated rats on either the training pellets (devalued condition) or homecage chow (valued condition) prior to brief non-reinforced test sessions after limited (sessions 5/6) and extended (sessions 17/18) PLA training. GT rats decreased conditioned responding under devalued relative to valued conditions after both limited and extended training, demonstrating they are sensitive to satiety devaluation regardless of the amount of PLA training. While ST rats were insensitive to satiety devaluation after limited training, their lever directed behavior became devaluation sensitive after extended training. To determine whether sign-tracking rats also displayed sensitivity to illness-induced outcome devaluation after extended training, we trained a separate cohort of rats in extended PLA and devalued the outcome with lithium chloride injections after pellet consumption in the homecage. ST rats failed to decrease conditioned responding after illness-induced outcome devaluation, while Non-ST rats (GT and intermediates) were sensitive to illness-induced outcome devaluation after extended training. Together, our results confirm devaluation sensitivity is stable in GT rats across training and devaluation approaches. Extended training unmasks devaluation sensitivity in ST rats after satiety, but not illness-induced devaluation, suggesting ST rats respond appropriately by decreasing responding to cues during state-dependent but not inference-based devaluation. The differences in behavioral flexibility across tracking groups and devaluation paradigms have translational relevance for the understanding state- vs. inference-based reward devaluation as it pertains to drug addiction vulnerability.

## Introduction

Pavlovian lever autoshaping (PLA) unveils distinct sign- and goal-tracking behaviors in rats. In this task, the insertion and retraction of a Pavlovian lever cue predicts food reward delivery. Rats are not required to interact with the lever; yet, sign-tracking (ST) rats preferentially approach and interact with the lever, while goal-tracking (GT) rats approach the food cup (Hearst and Jenkins, 1974; Boakes, 1977; Flagel et al., 2007). Studies suggest that ST and GT rats vary in the extent to which they attribute incentive motivational properties of cues (Flagel and Robinson, 2017). The lever cue is more attracting and reinforcing to ST than GT, and both natural and drug-associated cues invigorate instrumental responses to a greater degree in ST than GT, resulting in greater cue-induced relapse to drug-seeking (Tomie, 1996; Robinson and Flagel, 2009; Saunders and Robinson, 2010, 2013; Meyer et al., 2012; Yager and Robinson, 2013; Yager et al., 2015; Versaggi et al., 2016; Villaruel and Chaudhri, 2016). Sign-tracking in rats also predicts heightened drug relapse despite having to traverse an electrified barrier to seek and take drugs (Saunders and Robinson, 2013). We and others have shown that even prior to the drug experience, sign-trackers are inflexible, continuing to respond to cues when associated outcomes are devalued (Morrison et al., 2015; Nasser et al., 2015; Smedley and Smith, 2018). In contrast, goal-trackers flexibly adapt after outcome devaluation and are less susceptible to drug relapse when punishment is imposed (Saunders and Robinson, 2013; Nasser et al., 2015). Sign- and goal-tracking differences in adaptive behavior evident prior to and after drug experience highlight the utility of the sign- and goal-tracking model for understanding addiction vulnerability. Despite the trait-like qualities often attributed to ST and GT, we and others have observed a shift towards lever-directed behaviors in GT rats with extended PLA training (Villaruel and Chaudhri, 2016; Bacharach et al., 2018). The increased lever approach observed in GT rats leads to our prediction that behavior of GT rats will become devaluation insensitive as they shift towards sign-tracking behaviors.

Several studies reveal a dichotomy between GT and ST behaviors in devaluation sensitivity. Outcome devaluation by lithium chloride (LiCl)-induced conditioned taste aversion (CTA) decreases conditioned responding in GT and intermediate rats, while failing to reduce conditioned responding in ST rats (Nasser et al., 2015). The failure of ST to appropriately reduce responding to cues after devaluation is evident after both after light-food and lever-food Pavlovian conditioning (Morrison et al., 2015; Nasser et al., 2015). Satiety-induced devaluation similarly results in the flexibility of goal-tracking, but not sign-tracking behaviors in rats (Patitucci et al., 2016) and humans (De Tommaso et al., 2017). In contrast, sign-tracking behaviors are sensitive to satiety and illness-induced devaluation in other studies (Cleland and Davey, 1982; Derman et al., 2018). The differences in sign-tracking devaluation sensitivity between studies may be due to differences in the amount of training prior to devaluation procedures. Insensitivity to devaluation has been observed in ST rats after limited training [<10 training sessions; Morrison et al., 2015; Nasser et al., 2015)], and some studies using extended training prior to devaluation [>10 training sessions; (Patitucci et al., 2016; Smedley and Smith, 2018)]. Yet other studies using extended training prior to devaluation report devaluation sensitivity of lever directed behaviors generally, and in ST rats specifically (Cleland and Davey, 1982; Derman et al., 2018). Regarding goal-tracking behaviors, Pavlovian food cup directed behavior remains sensitive to devaluation, independent of the amount of training (Holland, 1998). Together, these studies suggest the observed tracking-related differences in devaluation sensitivity may be experience dependent. Here, we sought to determine how the amount of Pavlovian training influences devaluation sensitivity in sign- and goal-tracking rats.

We have previously shown GT, but not ST, rats are sensitive to LiCl-induced outcome devaluation (Nasser et al., 2015). However, this procedure results in permanent CTA, which would limit our ability to test whether extended training causes GT rats to become devaluation insensitive as they shift towards sign-tracking behaviors. Thus, in Experiment 1, we tested the same GT and ST rats on satiety-specific outcome devaluation after limited (five sessions of training) and after extended PLA (>15 sessions of training). First, we sought to determine whether sating rats on the outcome associated with the Pavlovian lever cue would also produce devaluation sensitivity in GT rats, but not ST rats, after limited training. Next, we continued to train the rats in PLA to the point that GT rats display more ST than GT behaviors. After GT rats shifted towards lever-directed behavior, we tested all rats again in satiety-specific outcome devaluation. We predicted that GT rats would be sensitive to devaluation after limited training but become devaluation insensitive after extended training when they shift towards sign-tracking behaviors. This outcome would suggest the behavioral flexibility observed in GT rats might not reflect a stable trait, instead providing evidence for experience-dependent variation in flexibility. If instead, GT and ST rats remain consistent in their devaluation sensitivity after both limited and extended training, this would suggest that behavioral flexibility differences reflect stable GT and ST trait differences.

To extend our previous study that used illness-induced outcome devaluation, and to compare the results of Experiment 1 with prior PLA devaluation studies (Cleland and Davey, 1982; Morrison et al., 2015; Nasser et al., 2015; Derman et al., 2018), in Experiment 2 we examined devaluation sensitivity using LiCl-induced outcome devaluation after extended training. Devaluation by satiation tests rats while they are in a sated state and probes approach immediately after *ad libitum* consumption of the training pellet, which requires little to no inference about the current value of the outcome. The illness-induced outcome devaluation procedure we use, instead, probes approach in a distinct context from and days after the last pairing of training pellets and illness. Thus, our illness-induced devaluation procedure requires that rats adjust responding to the lever cue based on inference about the current value of the outcome. The outcome of the two experiments allowed us to determine whether GT and ST groups differ in devaluation sensitivity based on how they respond in (1) a sated state during test and (2) an inference-based state during test, temporally and contextually distinct from taste aversion.

## Materials and Methods

### Subjects

Male Long–Evans rats (Charles Rivers Laboratories, Wilmington, MA, USA; 250–275 g upon arrival; Experiment 1, *n* = 48 run as two separate cohorts; Experiment 2, *n* = 38 run as one cohort) were double housed and maintained on a 12 h light/dark cycle (lights on at 07:00). All behavioral training and testing were conducted during the light phase of the cycle. During acclimation, rats had *ad libitum* access to standard laboratory chow and water. After acclimation, rats were singly housed, food-restricted, and maintained at ~90% of their baseline body weight throughout the experiment. All behavioral experiments were performed in accordance to the “Guide for the Care and Use of Laboratory Animals” (8th edition, 2011, US National Research Council) and were approved by the University of Maryland, School of Medicine Institutional Animal Care and Use Committee (IACUC).

### Apparatus

Behavioral experiments were conducted in identical behavioral chambers (25 × 27 × 30 cm; Med Associates) located in a room different than the colony room. Each chamber was in an individual sound-attenuating cubicle with a ventilation fan. During PLA and devaluation probe tests, each chamber had one red house light (6 W) located at the top of the wall that was illuminated for the duration of each session. The opposite wall of the chamber had a recessed food cup (with photo beam detectors) located 2 cm above the grid floor. The food cup had an attached programmed pellet dispenser to deliver 45 mg food pellets (catalog# 1811155; Test Diet Purified Rodent Tablet (5TUL); protein 20.6%, fat 12.7%, carbohydrate 66.7%). One retractable lever was positioned on either side of the food cup, counterbalanced between subjects, 6 cm above the floor. Sessions began with the illumination of the red house light and lasted ~26 min.

### Experiment 1: Satiety-Induced Outcome Devaluation

#### Limited and Extended Pavlovian Lever Autoshaping

**Figure 1:**
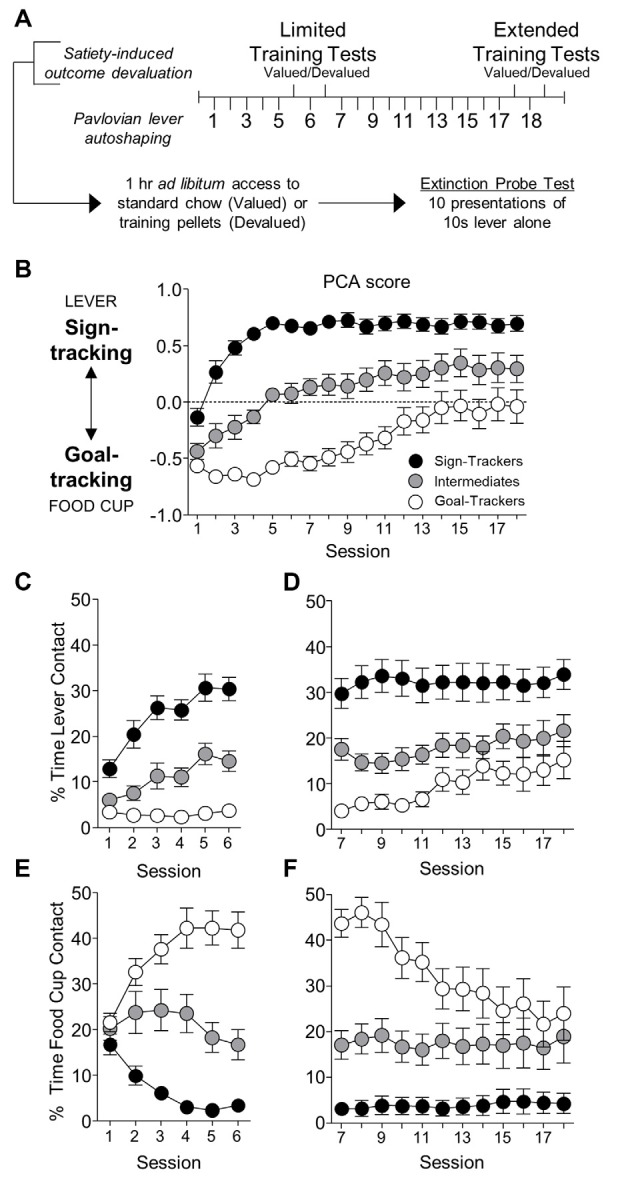
Experiment 1: lever- and food cup-directed behaviors during Pavlovian lever autoshaping (PLA). **(A)** Experimental timeline. We trained rats for five daily reinforced PLA sessions (1–5) to determine their tracking groups. We then tested rats using a within-subject satiety-induced outcome devaluation procedure after limited training. On test days we gave rats 1 h *ad libitum* access to standard chow (valued) or training pellets (devalued), counterbalanced, prior to 10 min probe tests that consisted of 10 CS presentations under extinction conditions. Between tests, we gave one reinforced PLA session. We gave rats 11 more reinforced PLA (7–17) sessions and repeated identical satiety-induced devaluation testing after extended training. **(B–F)** Data are mean ± standard error of the mean (SEM) for Pavlovian Conditioned Approach (PCA) scores **(B)**, percentage of time contacting the lever for limited **(C)** and extended PLA **(D)**, and percentage of time contacting the food cup for limited **(E)** and extended PLA **(F)**.

A summary of our experimental design can be found in Figure 1A. Prior to behavioral training, we exposed rats to a single 26 min magazine training session to reduce the novelty of the context and unconditioned stimuli. This session consisted of 25 trials in which two 45 mg pellets [Testdiet pellets (5TUL)] were delivered (0.5 s apart) on a VI 60 s schedule (50–70 s). The following day, we trained rats on daily, 26 min PLA sessions. Each session consisted of 25 presentations of a lever conditioned stimulus (CS+) occurring on a VI 60 s schedule (50–70 s). For each trial, the retractable lever was inserted for 10 s, then retracted, which was immediately followed with the delivery of two food pellets into the food cup, independent of contact with the lever or food cup. Following each training session, chow was provided to maintain rats at 90% of *ad libitum* body weight, and rats were transported back to the colony room.

#### Satiety-Induced Outcome Devaluation Procedures

As indicated in Figure 1A, after the 5th and 17th sessions of PLA, we gave rats two rounds of satiety-induced outcome devaluation tests. We sated half the rats by providing *ad libitum* access to the Testdiet training pellets (devalued condition), and the other half received standard chow (valued condition) in pre-habituated ceramic ramekins for 1 h in their homecage. Immediately, following consumption, we transported rats to the behavioral chambers for a 10 min lever autoshaping probe test under extinction conditions. Each probe test consisted of 10 presentations of the 10 s lever conditioned stimulus (VI 60 s, 50–70 s). After a day of rest, we retrained rats in a single reinforced PLA session. The next day we sated rats on the opposite condition (pellet or chow) prior to lever autoshaping probe test under extinction conditions. After extended PLA autoshaping (Figure 1A), we used identical procedures in the second round of satiety induced devaluation (pellet and chow). Notably, satiety conditions were reversed such that rats pre-fed on chow followed by pellets during limited testing received pellets followed by chow during extended testing and vice versa. This within-subject testing allowed us to measure responding in lever autoshaping during a sated state, either to the pellet outcome specifically associated with the lever (devalued) or generally sated on standard chow (valued) across conditioning.

#### Consumption Test of Specific Satiety

To confirm satiety was specific to the outcome on which rats were pre-fed, we gave one cohort a food choice test after all outcome devaluation testing was completed (cohort 1, *n* = 36; GT: *n* = 9; INT: *n* = 11; ST: *n* = 16). We provided rats with *ad libitum* access to either the training pellets or chow, counterbalanced for order, in ceramic ramekins for 1 h. Food was removed and replaced with two ceramic ramekins, one with 10 g of pellets and one with 10 g of chow for 30 min, after which we measured consumption of both outcomes. Two days later, we tested rats again after satiety with the opposite outcome.

### Experiment 2: Illness-Induced Outcome Devaluation

#### Extended Pavlovian Lever Autoshaping

We trained a separate cohort of rats through extended (17 sessions) training in PLA (Figure 4) prior to illness-induced devaluation. Magazine training and extended autoshaping were identical to Experiment 1, except rats were tested only after extended PLA training and not after limited training.

**Figure 2:**
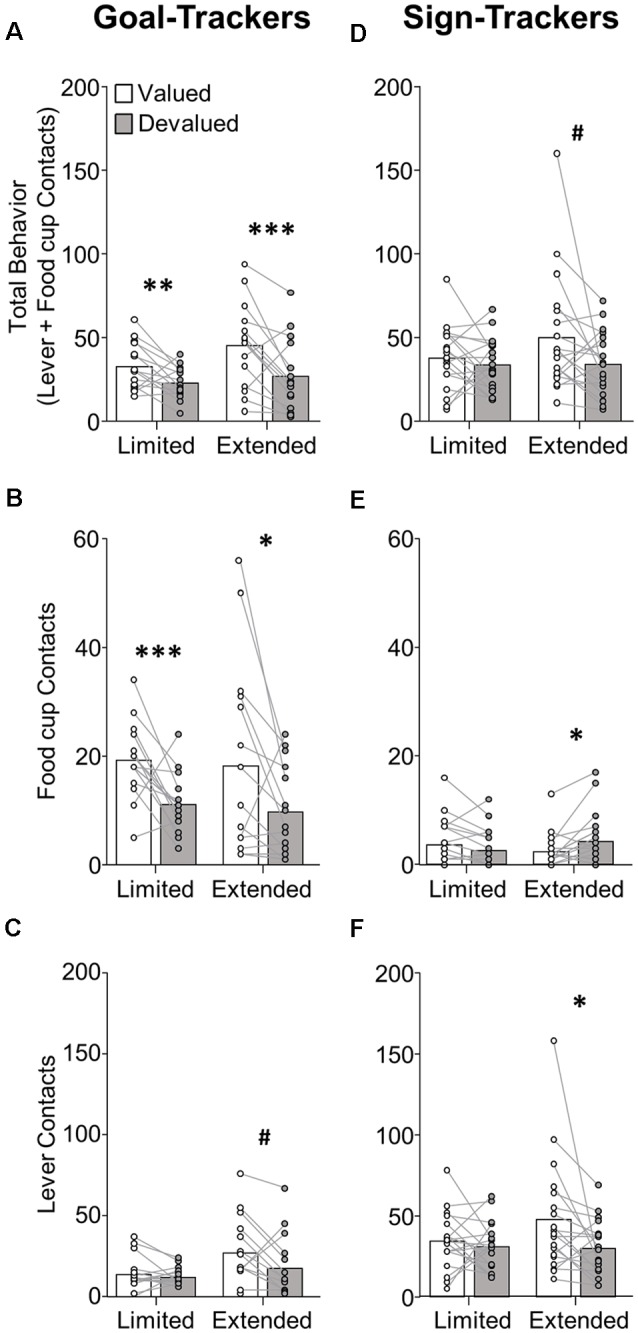
Experiment 1: performance during satiety-induced outcome devaluation probe test. Data show mean and individual data points for performance on devaluation probe test by goal-trackers (left column) and sign-trackers (right column), classified based on PCA scores after limited training. **(A–C)** Goal-trackers’ approach under valued (white bars) and devalued (gray bars) conditions during limited and extended satiety-induced outcome devaluation tests for **(A)** total behavior (sum of lever and food cup contacts), **(B)** food cup contacts, and **(C)** lever contacts. **(D–F)** Sign-trackers’ approach under valued (white bars) and devalued (gray bars) conditions during limited and extended satiety-induced outcome devaluation tests for **(A)** total behavior (sum of lever and food cup contacts), **(B)** food cup contacts, and **(C)** lever contacts. ^#^*p* = 0.051, *p < 0.05, **p < 0.025, ***p < 0.01.

**Figure 3:**
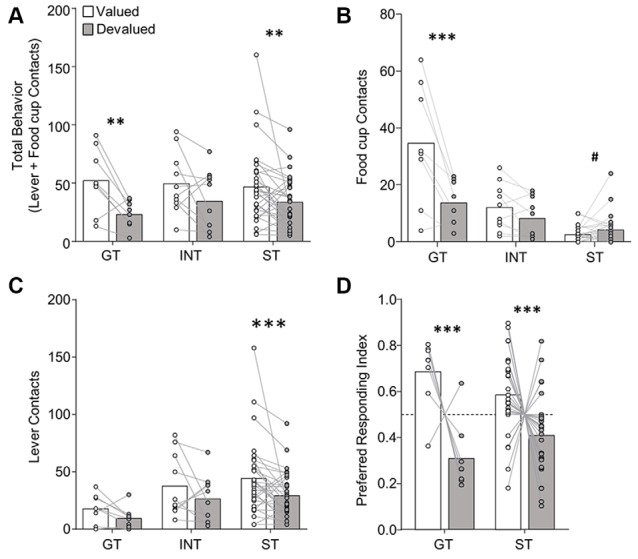
Experiment 1: performance during extended satiety-induced outcome devaluation probe test after reclassification of tracking groups based on PCA scores after extended training. Data show mean and individual data points. **(A)** Approach for reclassified GT, INT and ST under valued (white bars) and devalued (gray bars) conditions during extended satiety-induced outcome devaluation tests for **(A)** total behavior (sum of lever and food cup contacts), **(B)** food cup contacts, and **(C)** lever contacts. **(D)** Preferred responding index during valued relative to devalued conditions for goal-trackers (food cup contact) and sign-trackers (lever contact). Valued preferred responding index = valued preferred responding/(valued + devalued preferred responding). Devalued preferred responding index = devalued preferred responding/(valued + devalued preferred responding). A Preferred responding index value of 0.5 reflects similar responses during valued and devalued conditions. ^#^*p* = 0.056, **p < 0.025, ***p < 0.01.

**Figure 4:**
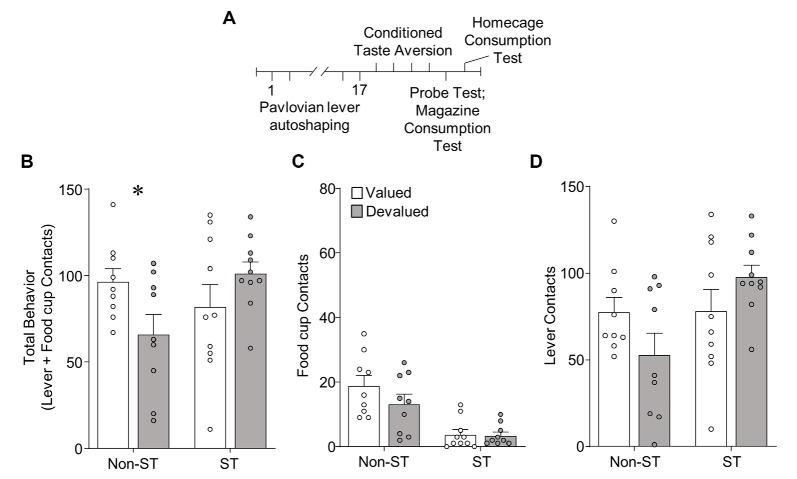
Experiment 2: performance during illness-induced outcome devaluation probe test after extended PLA. **(A)** Experimental timeline. We trained rats on 17 sessions of PLA. We split rats into valued (unpaired) and devalued (paired) groups gave 4 days of conditioned taste aversion (CTA) training in the rats’ homecages. After the last day of CTA, we conducted a 10 min probe test that consisted of 10 CS presentations under extinction conditions. Approximately 3 h after the probe test we gave rats 10 min access to 50 training pellets in the magazine of the conditioning chamber. The next day we gave rats 10 min access to 100 training pellets in their homecage to confirm CTA. **(B–D)** Effect of illness-induced devaluation on **(B)** total behavior (sum of lever and food cup contacts), **(C)** food cup contacts, and **(D)** lever contacts for Non-sign-tracking (Non-ST- made up of GT and INT rats) valued and devalued groups, left; and sign-tracking (ST) valued and devalued groups, right. Data are mean ± SEM and show individual data points. *p < 0.05.

#### Conditioned Taste Aversion Training

After extended PLA, we devalued the training pellets using a CTA procedure that took place in rats’ homecages over 4 days. Matching groups based on rats’ lever and food cup directed behavior during training, we divided rats into devalued (*n* = 19 pellet/LiCl paired) and valued (*n* = 19 pellet/LiCl unpaired) groups. Prior to the CTA procedure, we habituated all rats to the ceramic ramekins used to present the food pellets during CTA. On days 1 and 3 of CTA, we gave devalued rats 10 min access to 100 pellets in ceramic ramekins in their homecage followed immediately by lithium chloride (LiCl) injection (0.3 M, 5 ml/kg, i.p.), while we gave valued rats only the LiCl injection. On days 2 and 4, we gave valued rats 10 min access to 100 pellets in ceramic ramekins in their homecage, while devalued rats received no treatment. Across 4 days of CTA, we exposed all rats to training pellets and LiCl-induced gastric malaise; however, only the devalued group experienced pairings of training pellets with illness, while the valued group received explicit temporal separation of the training pellets and illness. We gave all rats standard homecage chow based on 90% body weight with compensation for CTA pellet consumption ~6 h after pellet and/or injections each day to prevent association of LiCl-induced illness with homecage chow.

#### Outcome Devaluation Probe Test

After the last day of CTA procedure, we conducted an outcome devaluation test under extinction conditions (Figure 4A). This 10 min lever autoshaping probe test consisted of 10 presentations of the 10 s lever conditioned stimulus (VI 60 s, 50–70 s) with no pellet delivery, identical to the probe test in Experiment 1. Approximately 3 h after the probe test, we gave rats a pellet consumption test in the conditioning chambers to assess generalization of the CTA from the homecage to conditioning chamber. We gave rats 10 min access to 50 training pellets that were placed in the magazine of the chambers, and we recorded the number of pellets consumed. The following day we confirmed CTA to the pellets in the homecages. We gave all rats 10 min access to 100 pellets in the ramekins in their homecage and recorded the number of pellets consumed.

### Behavioral Measurements

During the PLA and devaluation probe tests, we collected and recorded four behavioral measurements during the 10 s CS (lever) period. For both food cup contacts and lever contacts, the total number of contacts and total duration of response (% time) were automatically recorded for all sessions. The latencies to first contact the lever and food cup during the CS for each trial were also automatically recorded. On trials in which contact did not occur, a latency of 10 s was recorded. For each session the lever or food cup probabilities were calculated by determining the number of trials that the lever or food cup response was made, divided by the total number of trials in the session.

We used a Pavlovian Conditioned Approach (PCA) analysis (Meyer et al., 2012) to determine sign-, goal- and intermediate tracking groups. The PCA score quantifies the variation between lever-directed (sign tracking) and food cup-directed (goal tracking) behaviors. Each rat’s PCA score is the average of three difference score measures (each ranging from −1.0 to +1.0) including: (1) preference score; (2) latency score; and (3) probability score. The preference score is the duration of lever contacts during the CS minus the duration of food cup contacts during the CS, divided by the sum of these two measures. The latency score is the average latency to make a food cup contact during the CS minus the latency to lever contact during the CS, divided by the duration of the CS (10 s). The probability score is the probability of a lever contact minus the probability of a food cup contact observed across trials in the session. We determined tracking groups by averaging PCA scores during training sessions 4 and 5. Sign-tracking PCA scores range from +0.33 to +1.0, goal-tracking PCA scores range from −0.33 to −1.0, and intermediate group PCA scores range from −0.32 to +0.32.

For the devaluation tests, we examined total behavior, which is the sum of food cup and lever contacts during the 10 s CS period, and we also present these measures separately. We also examined preferred responding—food cup contact for goal-trackers and lever contact for sign-trackers. To account for differences in number of lever and food cup contacts, we examined rats’ preferred responding during valued relative to devalued conditions using the following equations: valued preferred responding index = valued preferred responding/(valued + devalued preferred responding) and devalued preferred responding index = devalued preferred responding/(valued + devalued preferred responding). A preferred responding index of 0.5 reflects similar responses during valued and devalued conditions.

We recorded the amount (in grams) of pellets and chow consumed during the hour of satiety prior to all outcome devaluation probe tests and during all the choice consumption tests. For illness-induced devaluation procedures, we recorded the total number of pellets consumed during the CTA procedures and test sessions.

### Statistical Analysis

Data were analyzed using SPSS statistical software (IBM v.25) with mixed-design repeated-measures ANOVAs. Significant main effects and interactions (*p* < 0.05) were followed by *post hoc* paired samples or independent *t*-tests when applicable. The between- and within-subject factors and dependent measures within each statistical analysis are described in the corresponding results section.

## Results

### Experiment 1: Satiety-Induced Devaluation

#### Pavlovian Lever Autoshaping

We first examined how lever- and food cup- directed behaviors change across training in sign-, goal-, and intermediate tracking rats. The experimental timeline is shown in Figure 1A. In Table 1 we summarize mixed ANOVA (Tracking × Session) main effects and interactions from lever autoshaping training session analyses for each composite measure that makes up the PCA score. In summary, the PCA scores for reinforced lever autoshaping sessions 1–18 are shown for three tracking groups in Figure 1B. Notably, we observed a significant main effect of Session (*F*_(17,765)_ = 36.75, *p* < 0.001) and a Tracking by Session interaction for PCA scores (Figure 1B; *F*_(34,765)_ = 4.84, *p* < 0.001), indicating that the behavior of sign-, goal-, and intermediate tracking rats is differentially affected by experience in lever autoshaping. When looking specifically in sign-tracking (ST) rats, lever directed behavior emerged during training sessions 1–6 (Figure 1C, Supplementary Table S1), indicated by a main effect of Session (ST lever; *F*_(5,90)_ = 14.18, *p* < 0.001), and like all other behaviors measured, remained remarkably stable across extended training sessions 7–18 (Figure 1D, Supplementary Table S2, *F*’s < 1.5, *p*’s > 0.1). Goal-trackers’ (GT) food cup behavior increased across training sessions 1–6 (Figure 1E, Supplementary Table S1), indicated by a main effect of Session (GT food cup; *F*_(5,70)_ = 12.06, *p* < 0.001). As extended training sessions 7–18 progressed, GT rats spent more time engaged with the lever (GT lever, Session main effect; *F*_(11,154)_ = 4.55, *p* < 0.001; Figure 1D), and less time at the food cup (GT food cup, Session main effect; *F*_(11,154)_ = 13.20, *p* < 0.001; Figure 1F). This shift is confirmed by analysis in GT rats, including Response (lever, food cup) and Session (7–18) as factors, for which the critical interaction (*F*_(11,154)_ = 11.93, *p* < 0.001) indicates that GT rats’ shift towards lever-directed responding and away from food cup responding during extended training (Supplementary Table S2). This shift during extended lever autoshaping is also evident in analysis of GT’s positive shift in PCA scores during sessions 7–18 (Session: *F*_(11,154)_ = 10.24, *p* < 0.001; Figure 1B), and each of the PCA composite measures summarized in Supplementary Table S2, including slower latency to first food cup contact (Supplementary Figure S1A), faster latency to first lever contact (Supplementary Figure S1B), reduced food cup contact probability (Supplementary Figure S1C), and increased lever contact probability (Supplementary Figure S1D). GT’s shift towards sign-tracking is consistent with our previous report (Bacharach et al., 2018). The analyses of approach behavior in intermediate (INT) rats are reported in Supplementary Materials.

**Table 1:** Repeated measures ANOVAs for Pavlovian lever autoshaping across all tracking groups.

		Lever									
Effect	Degrees of Freedom	Contact		Count		Latency		Probability			
		*F*	*p*	*F*	*p*	*F*	*p*	*F*	*p*		
Session	(17,765)	26.61	<0.001	14.35	<0.001	50.13	<0.001	24.20	<0.001		
Tracking Group	(2,45)	17.22	<0.001	27.42	<0.001	12.99	<0.001	13.40	<0.001		
Session × Tracking group	(34,765)	1.60	0.017	1.75	0.005	4.55	<0.001	4.91	<0.001		
		Food Cup								PCA	
Effect	Degrees of Freedom	Contact		Counts		Latency		Probability		Counts	
		*F*	*p*	*F*	*p*	*F*	*p*	*F*	*p*	*F*	*p*
Session	(17,765)	0.40	0.99	4.74	<0.001	17.00	<0.001	15.85	<0.001	36.75	<0.001
Tracking Group	(2,45)	22.29	<0.001	33.24	<0.001	33.85	<0.001	31.90	<0.001	52.82	<0.001
Session × Tracking group	(34,765)	2.68	<0.001	5.86	<0.001	5.03	<0.001	4.84	<0.001	4.83	<0.001

#### Devaluation Tests

Next, we investigated the effects of limited and extended lever autoshaping training on satiety-induced outcome devaluation in GT and ST rats. After replicating our prior observation that GT rats shift towards sign-tracking responses with extended training, we set out to test our *a priori* hypothesis that extended training would make GT rats devaluation insensitive. The primary predictions for GT rats are (1) with limited training, GT rats are devaluation sensitive, replicating effects we and others have previously reported (Morrison et al., 2015; Nasser et al., 2015; Patitucci et al., 2016) and (2) with extended training, GT become devaluation insensitive. Based on prior reports of devaluation insensitivity in ST rats, we hypothesized that after both limited and extended training ST rats would be insensitive to devaluation. To directly test these two *a priori* hypotheses, we separately examined satiety-induced outcome devaluation effects in GT and ST rats.

##### GT Show Devaluation Sensitivity After Limited and Extended Training in Lever Autoshaping

Devaluation performance for GT rats after limited and extended training is shown in Figures 2A–C. Due to GT rats’ shift in approach from food cup to lever across conditioning, we first examined effects of satiety devaluation on total approach behavior (sum of lever and food cup contacts) using repeated-measures ANOVA with factors of Phase (limited, extended) and Devaluation Condition (valued, devalued). This revealed a main effect of Devaluation (*F*_(1,14)_ = 27.96, *p* < 0.001), expressed as a difference in total approach behavior between the valued and devalued condition during limited (*p* = 0.019) and extended (*p* = 0.006) tests (Figure 2A). Based on our consistent observation that GT shift from primarily food-cup behaviors to increasingly more lever directed behavior during extended training (Figure 1, Supplementary Figure S1; Bacharach et al., 2018), we sought to examine the devaluation sensitivity of each approach response across conditioning. We incorporated Response (lever, food cup) factor into the analysis above maintaining devaluation effect reported above and revealing a marginally significant Phase by Response interaction (*F*_(1,14)_ = 3.80, *p* = 0.072). These results suggest that GT rats maintain devaluation sensitivity throughout training. We aimed to confirm that the effects of devaluation were evident in both food cup and the lever approach. Analysis of food cup contacts alone in GT rats revealed main effect of Devaluation (*F*_(1,14)_ = 20.39, *p* < 0.001), and *post hoc*’s confirmed devaluation sensitivity of food cup contacts during the limited (*t*_(14)_ = 3.713, *p* = 0.002) and extended tests (*t*_(14)_ = 2.305, *p* = 0.037; Figure 2B). Analysis of lever contacts alone in GT rats revealed a marginal main effect of Phase (*F*_(1,14)_ = 4.28, *p* = 0.058) but no interaction (*F* < 2.1, *p* > 0.1), with *post hoc*’s confirming marginal devaluation sensitivity of lever approach only after extended training (*t*_(14)_ = 2.134, *p* = 0.051; Figure 2C). These results suggest that despite a shift in response from food cup to lever directed behavior across conditioning, GT rats use the current value of the outcome to flexibly guide both forms of approach.

##### ST Show Devaluation Sensitivity Only After Extended Training in Lever Autoshaping

Devaluation performance for ST rats after limited and extended training is shown in Figures 2D–F. We examined the effects of satiety devaluation on total approach behavior using repeated-measures ANOVA with factors of Phase (limited, extended) and Devaluation (valued, devalued). This revealed marginal main effects of Devaluation (*F*_(1,18)_ = 3.92, *p* = 0.063) and Phase (*F*_(1,18)_ = 4.15, *p* = 0.057). *Post hoc* analysis revealed a marginal devaluation sensitivity in ST only after extended training (*p* = 0.051). We incorporated Response (lever, food cup) factor into the analysis above and found a Response by Devaluation interaction (*F*_(1,18)_ = 4.61, *p* = 0.046) and a Phase by Response by Devaluation interaction (*F*_(1,18)_ = 5.15, *p* = 0.036), suggesting devaluation sensitivity in ST is carried by a specific response and varies based on the extent of training. *Post hoc* analyses revealed that after extended training ST rats respond more at the lever during valued relative to devalued tests (*t*_(18)_ = 2.277, *p* = 0.035; Figure 2F), suggesting that lever responding becomes more sensitive to devaluation with extended training. While there is very little food cup responding in ST rats, we observed a devaluation effect in the opposite direction for food cup contacts during the extended test (devalued > valued, *p* = 0.026; Figure 2E), which may account for the marginally significant devaluation sensitivity of ST when examining total approach behavior. Overall, these results suggest ST rats that are insensitive to devaluation after limited training become more sensitive to devaluation after extended Pavlovian training. This emerging devaluation sensitivity in ST rats is specific to lever responding.

##### Intermediates

The analyses up to this point were based on our *a priori* hypotheses about GT and ST rats. We also analyzed devaluation test data for intermediate rats that approach the food cup and lever at comparable levels (Supplementary Figure S2). We present parallel analyses on intermediate rats’ devaluation test data in the Supplementary Materials. Intermediate data is also included in the next analysis that compares devaluation sensitivity considering tracking group reclassification after extended training.

##### Devaluation Sensitivity After Extended Training Accounting for Tracking Group Shifts

In the interest of comparing our results with that of prior studies, we analyzed the extended devaluation test data after reclassification of tracking group due to the shift in PCA scores that occurred during extended training (Figure 3). We calculated PCA scores for session 17, which was the last session of training prior to extended devaluation testing and resulted in 8 GT, 10 INT, and 30 ST. We analyzed the approach behavior during extended devaluation tests using a repeated-measures ANOVA including between-subject factor of Tracking group (GT, INT, ST) and within-subject factors of Devaluation (valued, devalued) and Response (lever, food cup). We observed a main effect of Devaluation (*F*_(1,45)_ = 15.58, *p* < 0.001), and a Devaluation by Response by Tracking group interaction (*F*_(2,45)_ = 3.41, *p* < 0.05). *Post hoc* analysis revealed reclassified GT rats were devaluation sensitive on food cup contacts (*t*_(7)_ = 3.80, *p* < 0.01; Figure 3B) and reclassified ST were devaluation sensitive on lever contacts (*t*_(29)_ = 2.77, *p* < 0.01; Figure 3C). When we analyzed the data with only reclassified GT and ST groups (excluding reclassified INT), we observed the same statistical outcomes as reported above (same significant main effects and interactions). To eliminate the difference in response levels between GT and ST groups, we examined each rat’s preferred response (GT food cup, ST lever) data for the late outcome devaluation test session (see figure legend and methods for preferred response index calculation; a value of 0.5 means rats responded equally during the valued and devalued tests). After extended training, we observed a main effect of Devaluation (*F*_(1,36)_ = 17.29, *p* < 0.001; Figure 3D), but no Devaluation by Tracking group interaction (*F* < 2.5, *p* > 0.1), confirming devaluation sensitivity of GT and ST after extended training. Altogether, Experiment 1 results suggest regardless of when tracking group classification occurs, GT rats are devaluation sensitive, and after extended training ST rats become sensitive to satiety-induced outcome devaluation.

##### Satiety and Devaluation Choice Test

Prior to the devaluation tests sessions, we found no difference in the amount of food consumed between tracking groups during the satiation hour (*F* < 0.1, *p* > 0.8). To confirm the devaluation of the sated food, we gave one cohort of rats a post-satiety choice test. Rats consistently chose to consume the food they were not sated on, as indicated by the main effect of Choice (*F*_(1,23)_ = 356.62, *p* < 0.001). There were no Tracking main effects (*F* < 1.0, *p* > 0.4) or Tracking by Choice interactions (*F* < 1.0, *p* > 0.6), indicating ST and GT rats had a similar preference for the non-sated food during choice test. Because the satiety procedures for devaluation and choice tests were identical, we infer that satiety conditions going into devaluation probe tests were equivalent for both tracking groups.

### Experiment 2: Illness-Induced Outcome Devaluation

#### Pavlovian Lever Autoshaping

The experimental timeline is shown in Figure 4A. We gave rats 17 sessions of PLA followed by CTA training to the pellets and devaluation probe and consumption tests. The PCA scores for reinforced lever autoshaping sessions 1–17 are shown in Supplementary Figure S3A. Tracking group is based on PCA score classification during extended PLA session 17 to make our results more comparable to previous studies that examined illness-induced outcome devaluation after extended training. Similar to Experiment 1, we observed a significant main effect of Session (*F*_(16,576)_ = 41.77, *p* < 0.001) and a Tracking by Session interaction for PCA scores (Supplementary Figure S3A; *F*_(16,576)_ = 6.97, *p* < 0.001). We analyzed lever- and food cup-directed behaviors throughout PLA using repeated-measures ANOVA with a between-subject factor of Tracking group [Non-ST (GT and INT), ST] and within-subject factors of Response (lever, food cup) and Session (1–17; Supplementary Figures S3B,C). As expected, we observed a Response by Tracking group interaction (*F*_(1,36)_ = 32.23, *p* < 0.001), a Response by Session interaction (*F*_(16,576)_ = 15.01, *p* < 0.001), and a Response by Session by Tracking group interaction (*F*_(16,576)_ = 4.63, *p* < 0.001), indicating that behavior of Non-ST (GT and INT) and ST rats is differentially affected by experience in lever autoshaping, similar to Experiment 1.

#### Devaluation Testing

In Experiment 2, we gave rats the same amount of extended PLA as in Experiment 1, before homecage LiCl-induced CTA procedures [devalued (paired), valued (unpaired)]. We examined approach in a single outcome devaluation test under extinction conditions. Outcome devaluation performance is shown in Figure 4. We analyzed devaluation total approach behavior using repeated measures ANOVA including between-subject factors of Tracking group [Non-ST (GT and INT), ST] and Devaluation (valued, devalued). We performed *post hoc* analysis based on a Devaluation by Tracking group interaction (*F*_(1,34)_ = 6.22, *p* < 0.05), which revealed Non-ST (GT and INT) rats were sensitive to devaluation (*t*_(16)_ = 2.23, *p* < 0.05; Figure 4A). In contrast, ST rats did not show sensitivity to devaluation on total behavior (*p*’s > 0.1; Figure 4A). We present Non-ST and ST food cup and lever data in Figures 4C,D, respectively. Incorporating Response as a factor in the analysis revealed the expected main effect of Response (*F*_(1,34)_ = 144.17, *p* < 0.001), and Response by Tracking group interaction (*F*_(1,34)_ = 10.05, *p* < 0.01), which are mainly driven by the group assignments and a greater level of lever compared to food cup contacts. We did not observe a Devaluation by Response by Tracking group interaction (*F* < 3.1, *p* > 0.05), suggesting a specific response did not carry the devaluation difference between Non-ST and ST groups. Altogether, results suggest Non-ST rats’ total approach behavior is sensitive to inference-based devaluation after extended training, consistent with our prior study (Nasser et al., 2015). In contrast, ST rats’ total approach is devaluation insensitive, consistent with our prior results using inference-based homecage LiCl-induced devaluation (Nasser et al., 2015).

#### Conditioned Taste Aversion Training and Consumption Tests

To confirm CTA, we analyzed the CTA data using a mixed model repeated measures ANOVA with between-subject factors of Devaluation group (valued, devalued) and Tracking group [Non-ST (GT and INT), ST] and within-subject factor of Trial (1, 2). We found main effects of Devaluation group (*F*_(1,34)_ = 38.07, *p* < 0.001) and Trial (*F*_(1,34)_ = 36.24, *p* < 0.001) and a Trial by Devaluation group interaction (*F*_(1,34)_ = 57.14, *p* < 0.001). Critically, there were no main effects of nor interactions with the Tracking group (*F*’s < 3.8, *p*’s > 0.05; Supplementary Figure S4A), suggesting the devalued groups that received pellet-LiCl pairings, developed a CTA to the pellets with no difference between tracking groups.

On the same day of the devaluation probe test, we performed a pellet consumption test in the experimental chamber to confirm aversion to the pellets in the training context. Using an ANOVA with between-subjects factors of Devaluation group (valued, devalued) and Tracking group [Non-ST (GT and INT), ST], we observed the expected main effect of Devaluation (*F*_(1,34)_ = 92.43, *p* < 0.001), but no main effect of Tracking group or interaction (*F*’s < 3.6, *p*’s > 0.05; Supplementary Figure S4B). The following day we performed a post-probe homecage consumption test and analyzed consumption data using an ANOVA with between-subject factors of Devaluation (valued, devalued) and Tracking group [Non-ST (GT and INT), ST]. We also observed a main effect of Devaluation (*F*_(1,34)_ = 714.86, *p* < 0.001), but no main effect of Tracking group or interaction (*F*’s < 3.5, *p*’s > 0.1; Supplementary Figure S4A). The consumption data indicate successful and strong CTA to the training pellets in the devalued groups that readily transfer to the experimental chambers and, importantly, no differences between tracking groups. Additionally, these results confirm behavioral differences observed during the devaluation probe test reflect differences in inference-based devaluation and not a result of differences in CTA training.

## Discussion

Using a within-subject satiety-induced outcome devaluation procedure, we replicated our previous findings that after limited Pavlovian training, GT rats are sensitive to outcome devaluation while ST rats are not (Nasser et al., 2015). This tracking-specific devaluation sensitivity has replicated across several studies, Pavlovian paradigms, and devaluation procedures (Nasser et al., 2015; Patitucci et al., 2016; Smedley and Smith, 2018). As GT rats increased lever-directed and decreased food cup-directed behaviors across extended training, we expected their behavior to become inflexible during outcome devaluation, similar to ST rats. However, GT rats remained devaluation sensitive for both lever- and food cup-directed behaviors after extended training. ST rats’ lever-directed behavior became sensitive to satiety-induced outcome devaluation after extended training in PLA. The results of Experiment 1 suggest GT rats remain behaviorally flexible, regardless of the amount of training, while ST rats become flexible with extended training. However, in Experiment 2, while a Non-ST group, made up of GT and intermediates rats, flexibly reduced approach after illness-induced outcome devaluation, ST rats inflexibly responded to lever cues after extended training. The differences in devaluation sensitivity of ST and Non-ST rats in Experiment 2 are consistent with our prior study using illness-induced outcome devaluation after limited training (Nasser et al., 2015).

The tracking-, training-, and devaluation procedure-dependent differences reported here may help to explain the inconsistencies in devaluation sensitivity of sign- and goal-tracking behaviors reported in prior studies. The strong influence of training duration is confirmed by the present within-subject measurement of satiety-induced devaluation sensitivity in ST rats. In Experiment 1, we found the same ST individuals that are insensitive early in training become sensitive to satiety devaluation with extended training. Conversely, we consistently find that ST rats are insensitive to illness-induced devaluation after limited Pavlovian conditioning (Nasser et al., 2015), and extended PLA training (Experiment 2), consistent with studies from other labs (Morrison et al., 2015). Yet, other studies using illness-induced outcome devaluation find the lever approach to be devaluation sensitive, suggesting other procedural differences may account for contrasting findings (Cleland and Davey, 1982; Derman et al., 2018).

The devaluation sensitivity of GT rats and associated food cup behaviors is consistent with other studies using limited training procedures (<10 sessions). These prior studies used LiCl-induced CTA to devalue outcomes associated with classic Pavlovian cues, including lights, tones, and levers (Holland, 1998; Morrison et al., 2015; Nasser et al., 2015). Consistently, we find GT behavior (food cup approach) is devaluation sensitive across devaluation procedures (satiety and LiCl) and amounts of conditioning. This is entirely consistent with the finding that classic Pavlovian auditory conditioning, which preferentially promotes food-cup behavior, is sensitive to devaluation independent of the number of CS-US pairings (Holland, 1977, 1998). The present study extends these findings, showing that GT rats, defined by their food cup approach, remain sensitive to devaluation with more CS-US pairings, despite a shift away from predominantly food cup-directed and towards lever-directed behaviors. This shift in goal-tracking towards sign-tracking has been reliably observed across labs using different reinforcers (Villaruel and Chaudhri, 2016; Bacharach et al., 2018; Derman et al., 2018). The present study adds an important psychological caveat to the goal-to-sign-tracking behavioral shift, which may appear maladaptive at face value, but remains adaptively sensitive to manipulations of outcome value.

The devaluation insensitivity of ST behavior that we observe after limited training is consistent with several studies that vary in the amount of training prior to outcome devaluation. Insensitivity to devaluation has been observed in ST rats after limited training (<10 training sessions; (Morrison et al., 2015; Nasser et al., 2015) and for lever-directed behaviors after more extended training (>10 training sessions; (Patitucci et al., 2016; Smedley and Smith, 2018).

The emergence of satiety-induced devaluation sensitivity of sign-tracking rats that we observe with extended training is consistent with other studies that also extensively conditioned rats prior to outcome devaluation (>10 training sessions). Notably, two such studies report devaluation sensitivity of lever directed behaviors generally, and specifically in ST rats (Cleland and Davey, 1982; Derman et al., 2018). Extensive training (>25 sessions) of CS-US pairings result in robust devaluation sensitivity of both lever- and food cup-directed behaviors after both illness- and satiety-induced outcome devaluation procedures (Cleland and Davey, 1982). Comparatively less extensive PLA training on two distinct levers (12 sessions of training on each lever contingency for a total of 24 training sessions) also results in robust devaluation sensitivity of lever-directed behaviors (during the cue) and magazine entries (post-cue; Derman et al., 2018). In contrast, after extended training we observe ST rats’ lever directed approach becomes devaluation sensitive but is accompanied by a small increase in their non-preferred food cup approach under devalued conditions. We expect the decreased preferred lever approach we observe in ST rats is primarily driven by the current value of the associated outcome. As a result of less lever interaction time, we postulate that the non-preferred food cup approach emerges, which may reflect response competition specifically under devalued conditions. Certainly, this emergence of food cup approach is not sensitive to the current value of the outcome. While we do not observe the emergence of a non-preferred lever approach in GT rats under devalued relative to valued conditions, others have (Morrison et al., 2015). This suggests the emergence of non-preferred responding under devalued conditions is not specific to GT or ST rats and is clearly not sensitive to the current value of the outcome. Notably, the amount of non-preferred responding we observe here after extended training in ST rats represents a very small fraction of overall approach behavior, which is largely driven by the preferred, devaluation sensitive, lever approach. Finally, in Experiment 2, after extended training we observed devaluation sensitivity in GT and intermediate rats (Non-ST), but not in ST rats after illness-induced outcome devaluation. The devaluation sensitive behavior of Non-ST rats was expressed as a decrease in total approach, and neither group showed an emergence of non-preferred responding in the devaluation condition. This difference in the emergence of non-preferred responding for satiety, but not LiCl devaluation suggests potentially divergent psychological processes that warrant further investigation. Altogether, the conflicting observations that sign-tracking and lever-directed behaviors are devaluation sensitive in some studies and insensitive in other studies suggests experience and other procedural differences may be key factors in experimental outcomes (Cleland and Davey, 1982; Patitucci et al., 2016; Derman et al., 2018; Smedley and Smith, 2018).

Notably, such studies have varied considerably in the devaluation approach (satiety, illness), context in which US was devalued (training, homecage, or novel context) and amount of CTA (limited, extensive US-LiCl pairings). Most notably, devaluation sensitivity of lever-directed behaviors is evident when CTA of the US is directly associated with the context in which the rats are trained and tested (Cleland and Davey, 1982; Derman et al., 2018). For CTA in these studies, rats consumed the US in the training context and then received injections of lithium chloride to induce gastric malaise. While the aim of CTA is for the gastric malaise to be associated solely with the US, the aversion induced by the gastric malaise could be generalized to the context itself, resulting in relevant contextual devaluation that influences later testing (Meachum, 1990; Boakes et al., 1997; Rodriguez et al., 2000; Limebeer et al., 2006). If rats are either in the sated state at test (Cleland and Davey, 1982; and Experiment 1 of the present study, but also see Patitucci et al., 2016) or are directly exposed to the training context with CTA (Cleland and Davey, 1982; Derman et al., 2018), then state-dependent or contextual associations may facilitate adaptive suppression of conditioned responding to cues at test (Bouton et al., 2019). There is arguably little inference required at the time of test when using satiety devaluation or CTA in training context procedures, in which devaluation occurs in a temporally contiguous way with exposure to the testing environment. In these cases, rats can use direct experience with the aversion to adaptively reduce responding to cues. Conversely, if CTA occurs independent of the training context, such as in the homecage or novel context, inference mediated by a representation of devalued outcome is required to adaptively reduce responding to associated cues [(Morrison et al., 2015; Nasser et al., 2015), Experiment 2 of the present study]. Additionally, both the context and amount of CTA (five rounds of US-LiCl pairings in training context in the Derman study) were greater in prior studies than in our Experiment 2 (two rounds of US-LiCl pairings in rats’ homecages). Altogether, we expect these contextual and CTA procedural differences contributed to the dissimilar findings between studies.

Here, in Experiment 1, we employed identical satiety devaluation manipulations after limited and extended training, thus controlling for devaluation procedure within-subject, and find that the same ST individuals that are insensitive early in training become sensitive to satiety devaluation with extended training. Our observations that ST rats are sensitive to satiety-, but not illness-, induced outcome devaluation after extended training suggests that ST rats are able to adjust behavior when tested in the devalued state (sated on pellet during testing in the chamber; Experiment 1). Specifically, this indicates ST rats can encode sensory-specific characteristics of the food they are sated on and relate this to the associated stimuli (i.e., lever) to appropriately respond during satiety-induced outcome devaluation, but only after extended PLA training. However, when an inference based on prior, contextually-distinct experience is required (Experiment 2), ST rats are unable to appropriately adjust behavior based on the current value of the outcome.

Instrumental devaluation studies pose a striking dissimilarity to Pavlovian studies regarding devaluation sensitivity after extended training. Instrumental responding, mediated by competing action-outcome and stimulus-response associations, transitions from goal-directed (devaluation sensitive) to habitual (devaluation insensitive) with extended instrumental experience (Adams, 1982; Dickinson et al., 1983; Blundell et al., 2003; Coutureau and Killcross, 2003; Killcross and Coutureau, 2003; Johnson et al., 2009; Parkes and Balleine, 2013). In contrast, Pavlovian behaviors remain sensitive to devaluation, independent of the amount of training (Holland, 1998). While fundamental differences between instrumental and Pavlovian associations likely explain these contrasting findings, the present study suggests individual differences in Pavlovian approach impact the expression of devaluation sensitivity. While we are careful in making parallels to the instrumental devaluation literature, the present study uses a Pavlovian task that results in sign-tracking (i.e., lever pressing), which resembles instrumental responses, but critically is not based on instrumental contingencies. In the instrumental literature, habit is operationally defined as insensitivity of instrumental actions to outcome devaluation, while goal-directed responding reflects the sensitivity of instrumental actions to outcome devaluation. Since rats on the GT end of the tracking continuum are consistently devaluation sensitive under all conditions tested in the current study, one might predict that GT rats would be more goal-directed than ST rats in instrumental settings, perhaps even independent of the amount of instrumental experience. Yet, other factors can impact instrumental devaluation sensitivity, including the amount of reinforcer experience (Adams, 1982), reinforcement schedules (continuous reinforcement, fixed interval/ratio, variable interval/ratio, etc.; Dickinson et al., 1983) and motivational state during training and testing (Balleine, 1992; Dickinson et al., 1995). The extent to which goal-directed vs. habitual responding emerges across conditioning in ST and GT rats requires instrumental devaluation procedures.

The current studies provide a behavioral framework to extend the investigation of individual behavioral and neurobiological differences in devaluation sensitivity, which we find depend on training history and devaluation approach. The individual and timing-dependent differences in devaluation sensitivity suggest potentially distinct and/or shifting neural mechanisms mediating the expression of behavior when outcome value changes. During limited training in PLA cue-evoked striatal dopamine release is evident in ST, but not GT rats, and intra-nucleus accumbens dopamine receptor antagonists reduce sign-, but not goal-tracking behaviors (Flagel et al., 2011; Saunders et al., 2013). Yet, the striatal dopamine release and dopamine-dependence associated with sign-tracking declines with extended training (Clark et al., 2013), at a time that we observe emerging devaluation sensitivity in ST rats. This raises the possibility that, after limited training, striatal dopamine signaling in ST rats masks devaluation sensitivity that is mediated by competing brain mechanisms.

The present study using male rats indicates the importance of considering tracking- and experience-dependent effects in inference-based and state-dependent devaluation. Future studies considering these factors in both sexes will elucidate more precisely defined contributions of dopamine and other brain circuits driving Pavlovian approach and devaluation sensitivity (Pickens et al., 2003; Coutureau et al., 2009; Johnson et al., 2009; Shiflett and Balleine, 2010; Parkes and Balleine, 2013; Zeeb and Winstanley, 2013; Hart et al., 2014; Parkes et al., 2015; Wassum and Izquierdo, 2015; Gourley et al., 2016; Mannella et al., 2016; West and Carelli, 2016; Izquierdo, 2017; Lichtenberg et al., 2017; Fisher et al., 2020). Such efforts are translationally relevant, as human studies report evidence for sign-tracking and individual variability in cue reactivity as well as devaluation sensitivity (Garofalo and di Pellegrino, 2015; Versace et al., 2016; De Tommaso et al., 2017; Pool et al., 2019). Particularly relevant is a recent study dissociating devaluation sensitive and insensitive responses during Pavlovian learning in humans (Pool et al., 2019). Future investigations of psychological and neurobiological differences that are evident prior to drug-experience will further our understanding of how sign- and goal-tracking differences relate to addiction vulnerability (Saunders and Robinson, 2010; Saunders et al., 2013; McClory and Spear, 2014; Versaggi et al., 2016; Pitchers et al., 2017; Valyear et al., 2017).

## Data Availability Statement

The raw data supporting the conclusions of this article will be made available by the authors, without undue reservation, to any qualified researcher.

## Ethics Statement

The animal study was reviewed and approved by University of Maryland, School of Medicine Institutional Animal Care and Use Committee.

## Author Contributions

DC conceived and supervised the project. SK, SB, JC, and DK acquired the data. SK analyzed the data. SK and DC designed the experiments, interpreted the data, and wrote the manuscript. All authors contributed to manuscript revision, read, and approved the submitted version.

## Conflict of Interest

The authors declare that the research was conducted in the absence of any commercial or financial relationships that could be construed as a potential conflict of interest.
