# Supplementary material for: Effects of Limited and Extended Pavlovian Training on Devaluation Sensitivity of Sign- and Goal-Tracking Rats
Source: Front Behav Neurosci. 2020 Feb 4;14:3. doi: 10.3389/fnbeh.2020.00003 (PMC7010919; doi:10.3389/fnbeh.2020.00003)
Supplement: Supplementary file 1 [file Table_1.docx]

***Supplemental Material***

**1 Supplementary Data**

**Intermediate Data**

**Pavlovian Lever Autoshaping.** We performed parallel analyses to main text on training data from intermediate rats that display both lever- and food cup-directed behaviors. Both lever and food cup behaviors emerged during training sessions 1-6 (Figure 1C, 1E), indicated by main effects of Session (Lever: *F* (5,65) = 5.59, *p* < 0.001; Food cup: *F* (5,65) = 2.64, *p* < 0.05). Similar to GT rats’ behavior across extended training sessions 7-18, intermediates increased time spent contacting the lever (Session main effect; *F* (11,143) = 2.51, *p* < 0.01, Figure 1D). Intermediates showed slower latency to first food cup contact (Session main effect; *F* (11,143) =3.23, *p* < 0.001, Figure S1A), but faster latency to first lever contact (Session main effect; *F* (11,143) = 1.87, *p* < 0.05, Figure S1B). Intermediates showed reduced food cup contact probability (Session main effect; *F* (11,143) =2.03, *p* < 0.05, Figure S1C) but no changes across training for time spent contacting food cup or probability of lever interaction (Figures 1F and S1D, *F*’s < 0.6, *p*’s > 0.8).

**Devaluation Tests.** Devaluation performance for INT rats (based on PCA scores after limited training) during limited and extended testing is shown in Figure S2A-C. We examined effects of satiety devaluation on total approach behavior using a repeated measures ANOVA with factors of Phase (limited, extended) and Devaluation (valued, devalued). This revealed a main effect of Devaluation (*F*(1, 13) = 8.85, *p* < 0.02, Figure S2A) but no effect of Phase or interaction (*F*’s < 1, *p*’s > 0.1). Post*-*hoc analysis that paralleled those performed for GT and ST rats revealed marginal devaluation sensitivity in INT after extended training (*p* = 0.069). In a similar analysis including Response (lever, food cup) as a factor, we found a main effect of Response (*F*(1,13) = 15.55, *p* < 0.01), due to INT rats contacting the lever more than the food cup, particularly after extended training, similar to GT rats.

**2 Supplementary Figures**

**
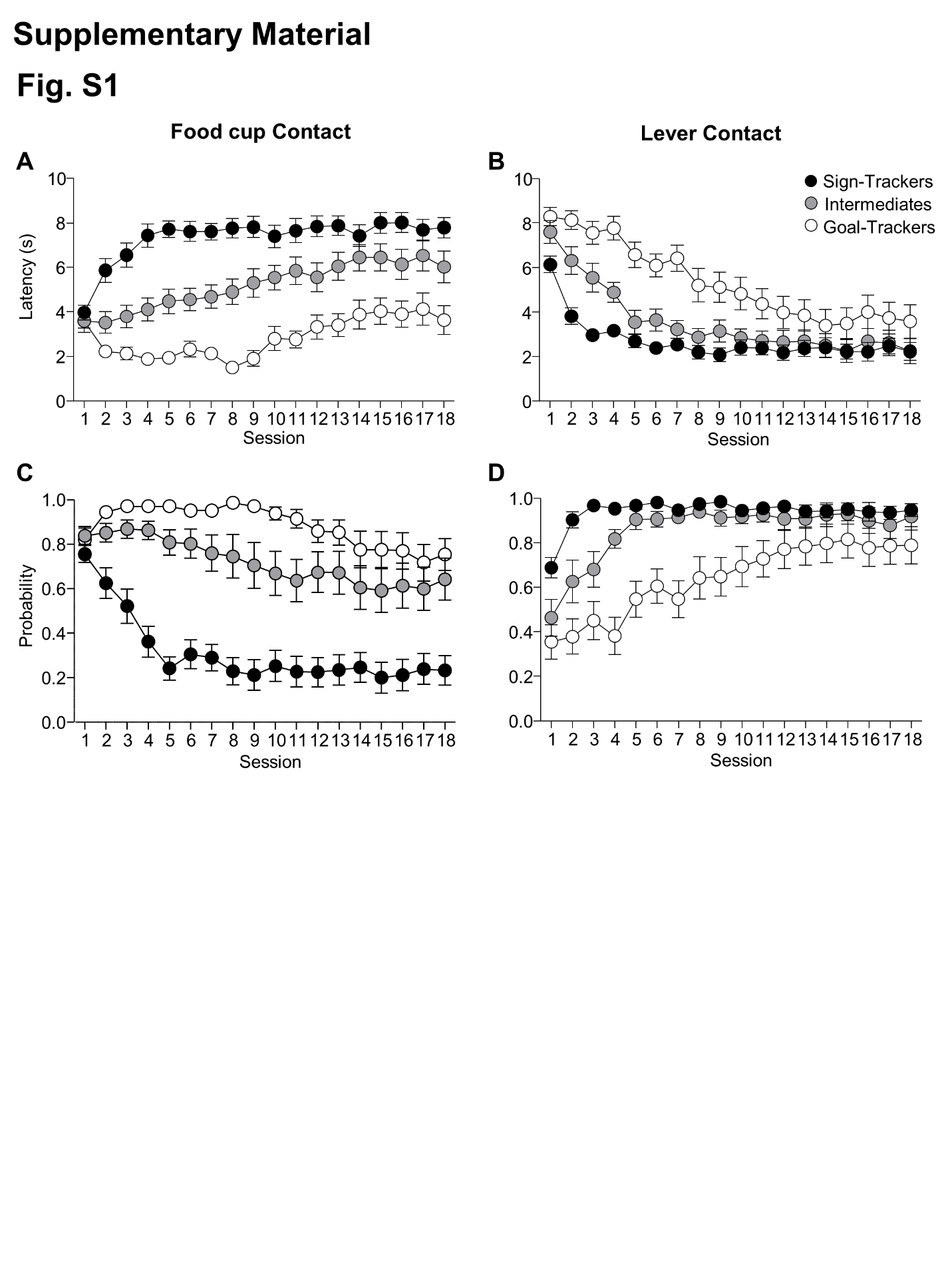
**

**Figure S1.** Experiment 1. Latency and probability data across all Pavlovian lever autoshaping (PLA) sessions. Data are mean ± standard error of the mean (SEM) for latency to contact (**A**) the food cup or (**B**) lever and probability of contacting (**C**) the food cup or (**D**) lever for sign-trackers, intermediates, and goal-trackers based on PCA scores after limited training.

**
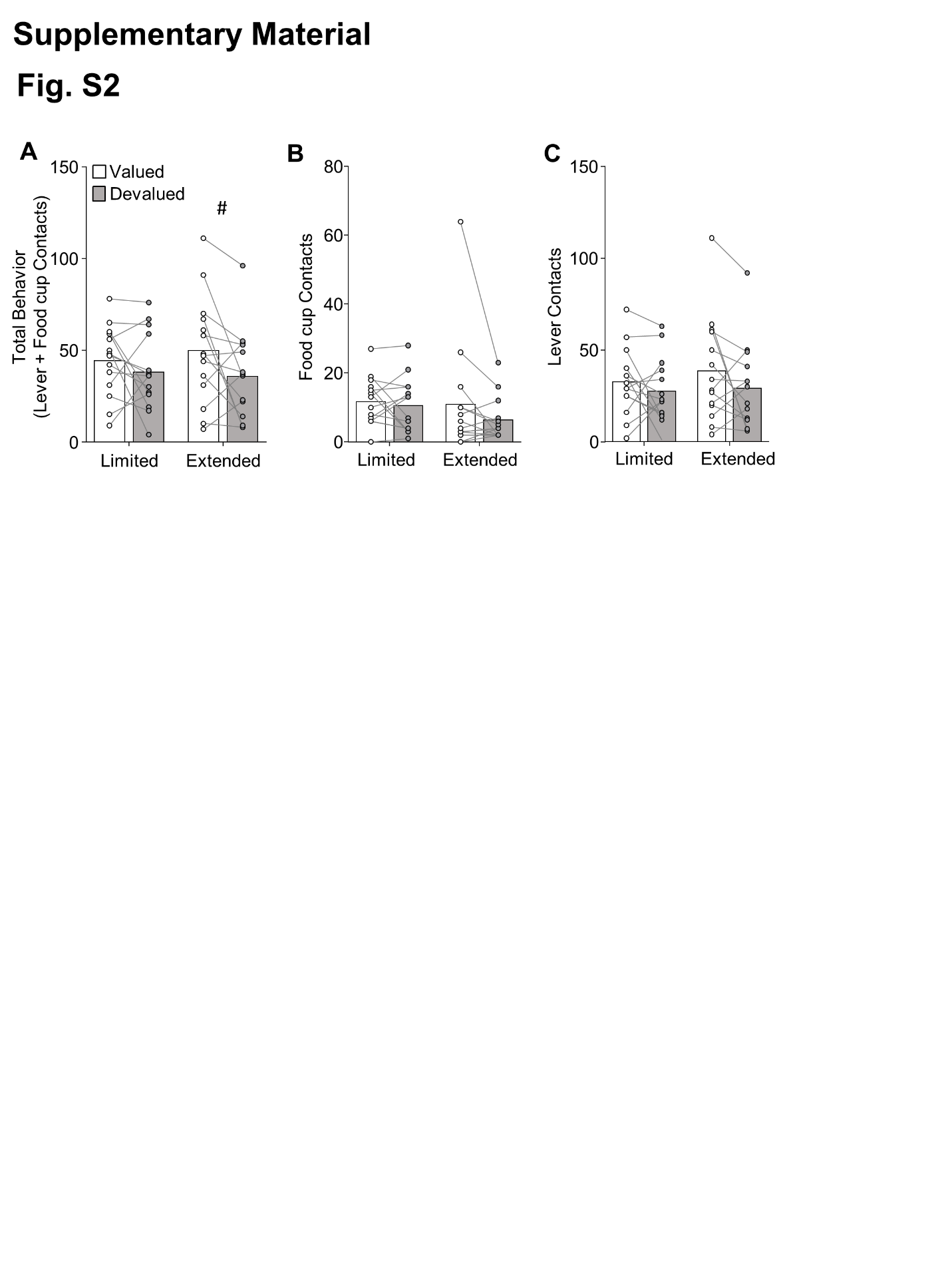
**

**Figure S2.** Experiment 1. Performance of intermediates (classified based on PCA scores after limited training) during satiety-induced outcome devaluation probe test. Data show and individual data points. Intermediates’ approach under Valued (white bars) and Devalued (grey bars) conditions during limited and extended satiety-induced outcome devaluation tests for (**A**) total behavior (sum of lever and food cup contacts), (**B**) food cup contacts, and (**C**) lever contacts. # *p* = 0.069.


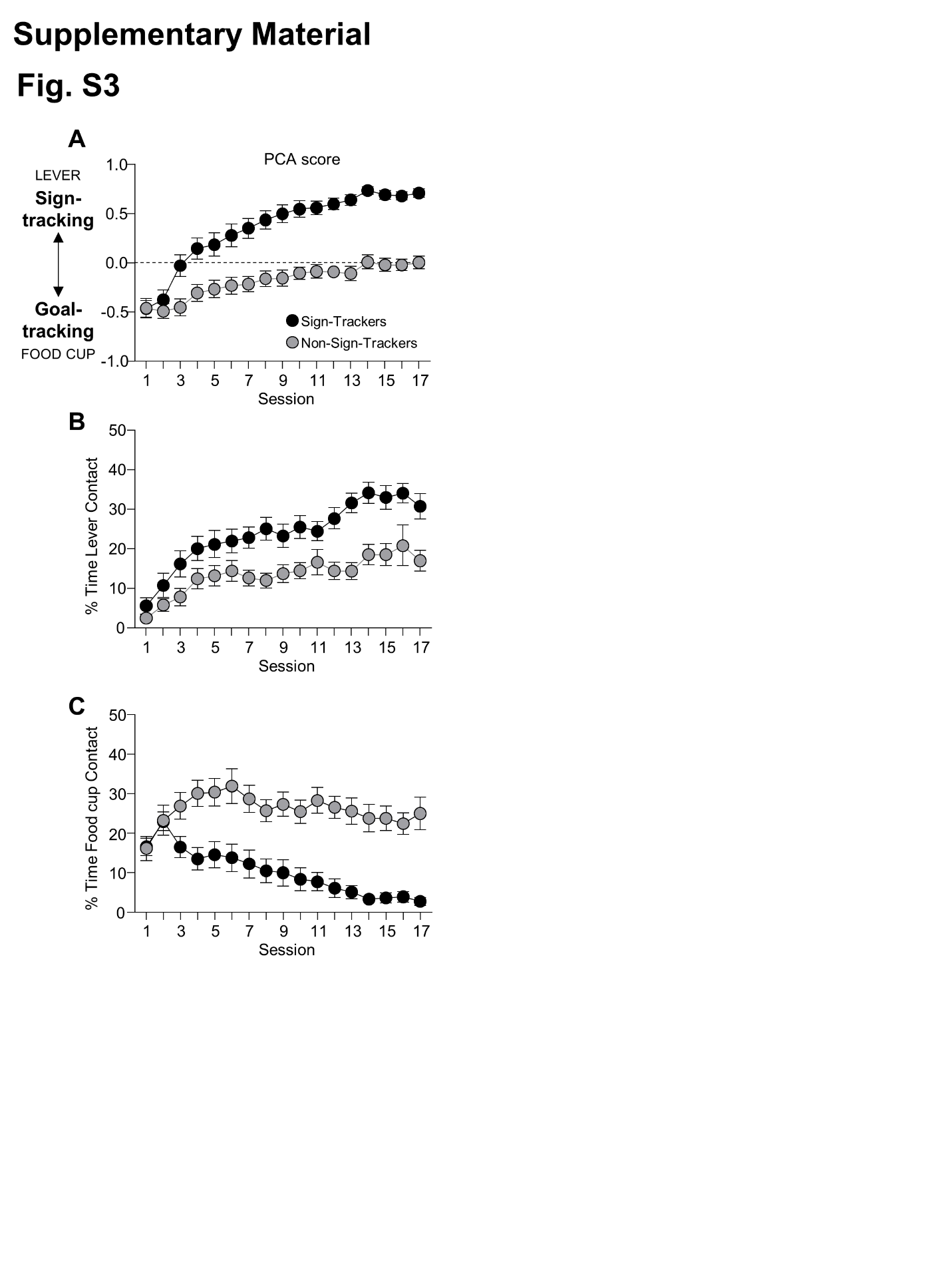


**Figure S3.** Experiment 2. Lever and food cup directed behaviors during Pavlovian lever autoshaping. **A**-**C** Data are mean ± standard error of the mean (SEM) for groups based on PCA scores after extended training. (**A**) PCA scores across PLA. Percentage of time contacting (**B**) the lever (**C**) and the food cup across PLA.


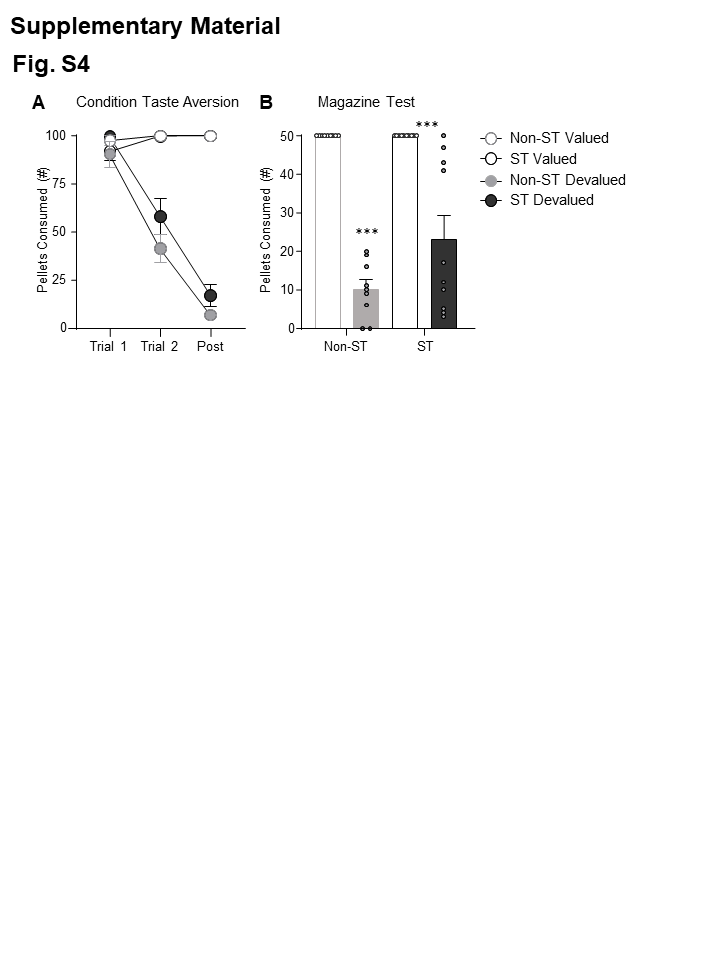


**Figure S4.** Experiment 2. Pellet consumption during (**A**) conditioned taste aversion training and post-probe test homecage test (Post) and (**B**) magazine consumption tests. Consumption for Non-Sign-Tracking (Non-ST- made up of GT and INT) valued (white) and devalued (light gray) groups and Sign-tracking (ST) valued (white) and devalued (dark gray) groups. Data are mean ± standard error of the mean (SEM) and show individual data points. *** *p* < 0.001 compared to Valued condition within same tracking group.

Supplementary Table 1. Repeated measures ANOVAs for early Pavlovian lever autoshaping for GT and ST: training sessions 1-6

|  |  | ST | | | | | | | | GT | | | | | | | |
| --- | --- | --- | --- | --- | --- | --- | --- | --- | --- | --- | --- | --- | --- | --- | --- | --- | --- |
| Effect | Degrees of Freedom | Contact | | Count | | Latency | | Probability | | Contact | | Counts | | Latency | | Probability | |
|  |  | *F* | *p* | *F* | *p* | *F* | *p* | *F* | *p* | *F* | *p* | *F* | *p* | *F* | *p* | *F* | *p* |
| Session | ST: 5,90  GT: 5,70 | 4.39 | 0.001 | 1.41 | 0.229 | 1.19 | 0.321 | 7.68 | < 0.001 | 7.12 | < 0.001 | 11.77 | < 0.001 | 10.06 | < 0.001 | 6.22 | < 0.001 |
| Response | ST: 1,18  GT: 1,14 | 189.46 | < 0.001 | 52.73 | < 0.001 | 47.41 | < 0.001 | 57.37 | < 0.001 | 42.20 | < 0.001 | 137.82 | < 0.001 | 94.37 | < 0.001 | 51.55 | < 0.001 |
| Session x Response | ST: 5,90  GT: 5,70 | 18.52 | < 0.001 | 24.25 | < 0.001 | 56.89 | < 0.001 | 41.14 | < 0.001 | 1.79 | 0.126 | 11.51 | < 0.001 | 4.73 | 0.001 | 3.39 | 0.008 |

p<0.05

Supplementary Table 2. Repeated measures ANOVAs for late Pavlovian lever autoshaping for GT and ST: training sessions 7-18

|  |  | ST | | | | | | | | GT | | | | | | | |
| --- | --- | --- | --- | --- | --- | --- | --- | --- | --- | --- | --- | --- | --- | --- | --- | --- | --- |
| Effect | Degrees of Freedom | Contact | | Count | | Latency | | Probability | | Contact | | Counts | | Latency | | Probability | |
|  |  | *F* | *p* | *F* | *p* | *F* | *p* | *F* | *p* | *F* | *p* | *F* | *p* | *F* | *p* | *F* | *p* |
| Session | ST: 11,198  GT: 11,154 | 0.88 | 0.561 | 0.60 | 0.831 | 0.40 | 0.956 | 0.95 | 0.494 | 3.76 | < 0.001 | 5.22 | < 0.001 | 1.76 | 0.065 | 1.11 | 0.360 |
| Response | ST: 1,18  GT: 1,14 | 77.44 | < 0.001 | 43.46 | < 0.001 | 130.15 | < 0.001 | 120.26 | < 0.001 | 1.66 | 0.219 | 15.53 | < 0.001 | 1.56 | 0.232 | 1.41 | 0.255 |
| Session x Response | ST: 11,198  GT: 11,154 | 1.21 | 0.284 | 0.33 | 0.979 | 1.52 | 0.125 | 1.62 | 0.096 | 2.24 | 0.015 | 11.93 | < 0.001 | 11.26 | < 0.001 | 8.47 | < 0.001 |

p<0.05
